# Feasibility of Ultra-Low-Dose CT for Bronchoscopy of Peripheral Lung Lesions

**DOI:** 10.3390/medicina56090479

**Published:** 2020-09-19

**Authors:** Jung Seop Eom, Geewon Lee, Jiyeon Roh, Hyun Sung Chung, Yeon Joo Jeong

**Affiliations:** 1 Department of Internal Medicine, Pusan National University School of Medicine, Busan 43241, Korea; ejspulm@gmail.com (J.S.E.); jiyeonroh@nate.com (J.R.); cattol@naver.com (H.S.C.); 2 Biomedical Research Institute, Pusan National University Hospital, Busan 43241, Korea; 3 Department of Radiology, Pusan National University School of Medicine, Busan 43241, Korea; rabkingdom@naver.com

**Keywords:** bronchoscopy, diagnosis, lung neoplasm, multisection computed tomography, radiation

## Abstract

*Background and objectives*: Thin-section computed tomography (CT) is essential for identifying small bronchi during bronchoscopy using radial endobronchial ultrasound. Some patients should receive an additional CT for a thin-section image. We performed a retrospective study with a prospectively collected database to identify the optimal radiation dose for thin-section CT during peripheral bronchoscopy. *Materials and Methods*: In total, 91 patients with peripheral lung lesions underwent thin-section CT (both standard CT as a reference and ultra-low-dose CT (ultra-LDCT)). The patients were randomly assigned to one of four groups according to the ultra-LDCT parameters: group 1 = 120 kVp, 25 mAs; group 2 = 100 kVp, 15 mAs; group 3 = 120 kVp, 5 mAs; and group 4 = 100 kVp, 5 mAs. Two radiologists and two physicians analyzed both the standard CT and ultra-LDCT. *Results*: The effective doses (EDs) of ultra-LDCT significantly differed among the four groups (median EDs were 0.88, 0.34, 0.19, and 0.12 mSv for groups 1–4, respectively; *p* < 0.001). Median differences in peripheral airway wall thickness were higher in group 4 than in other groups (differences in median wall thickness measured by two radiologists were 0.4–0.5 mm and 0.8–0.9 mm for groups 1–3 and group 4, respectively). Bronchus signs on ultra-LDCT in groups 1 and 2 were well correlated with those of the standard-dose CT (accuracies of two radiologists and two pulmonary physicians were 95–100%). *Conclusions*: Our results indicate that ultra-LDCT with ED of >0.34 mSv (ED of group 2) is feasible for peripheral bronchoscopy.

## 1. Introduction

The frequency of detecting peripheral lung lesions has increased with widespread use of low-dose chest computed tomography (CT) for lung cancer screening [1,2]. Although the accuracies of non-invasive imaging modalities (e.g., positron emission tomography and high-quality CT scans) have improved, a pathological examination remains the main method for a definitive diagnosis of lung cancer [3]. Transthoracic needle aspiration biopsies are generally considered to have good diagnostic performance; however, procedural-related complications, including pneumothorax, hemorrhage, and pleural seeding, can occur [4,5].

The technique of bronchoscopy for peripheral lung lesions, or so-called peripheral bronchoscopy, has substantially improved over the past few decades, such that it has become safer and provides an acceptable diagnostic yield [6,7,8]. Without any assistance from expensive navigation equipment (e.g., virtual bronchoscopy navigation and electromagnetic navigation bronchoscopy [9,10]), interpretation of thin-section CT before the bronchoscopic procedure, especially the identification of the bronchus sign, is the most critical step in planning peripheral bronchoscopy and biopsy. Although most patients have a CT performed for the diagnosis of the lung mass, some of these CT’s are not reconstructed with the section thickness to allow for recognition of the peripheral bronchi. These patients then need to get an additional thin section CT, which increases their radiation exposure. Our study aims to determine the minimum radiation dose that would allow the identification of peripheral bronchi on a thin section CT and hence keep the radiation exposure in these patients as low as possible.

## 2. Materials and Methods

### 2.1. Study Population

This retrospective study used a prospectively collected database to investigate the feasibility of 3D ultra-low-dose CT for bronchoscopy of peripheral lung lesions at Pusan National University Hospital (a university-affiliated, tertiary referral hospital in Busan, South Korea) between May 2017 and March 2018. During the study period, consecutive patients who received a chest CT scan for peripheral bronchoscopy were prospectively registered. Before collecting data of subjects, this study including randomization of subjects to different ultra-low-dose CT was approved by the Institutional Review Board of Pusan National University Hospital on 20 January 2020 (approval no. H-2001-005-087). Because of the retrospective nature of this study, the requirement for informed consent from each study subject was waived.

### 2.2. CT Protocol for Bronchoscopy of Peripheral Lung Lesions

All patients underwent both ultra-low-dose (pre-contrast enhanced) and standard-dose chest CT as reference (post-contrast enhanced) simultaneously. All CT examinations were performed during end-inspiratory breath-holding with patients in the supine position, using a 256-detector row CT (Revolution CT; GE Healthcare, Waukesha, WI, USA). Imaging parameters were held in helical imaging mode: 128 × 0.625 mm detector configuration and a 0.5 s rotation time. The standard-dose CT imaging parameters were: 120 kVp, tube current modulation with CTDIvol of ≤7 mGy (the tube current was between 100 and 250 mAs). All patients were randomly assigned to one of four study groups according to different ultra-low-dose CT imaging parameters, as follows: group 1 = 120 kVp, 25 mAs; group 2 = 100 kVp, 15 mAs; group 3 = 120 kVp, 5 mAs; and group 4 = 100 kVp, 5 mAs. Standard-dose CT images were reconstructed with filtered back projection, and ultra-low-dose CT images were acquired with 50% iterative reconstruction of strength. All reconstructed images were transferred to a dedicated workstation (Advantage Workstation 3.1; GE Healthcare) for analysis by radiologists.

### 2.3. CT Results and Image Analysis

To determine the estimated effective dose, the dose-length product was multiplied by a conversion coefficient of 0.014 mSv/mGy∙cm [11]. Two chest radiologists and two pulmonary physicians (one physician has 5 years of experience in peripheral bronchoscopy and the other has 2 years) evaluated the standard and ultra-low-dose CT scans for each patient. At first, two radiologists and two pulmonary physicians reviewed the ultra-low-dose CT scans, which blinded the assignment of each CT group; then, the standard CT scans were evaluated independently at least 2 weeks later as reference to compare ultra-low-dose CT. 

Objective image noise, wall thickness, and wall-area ratio of the bronchus leading to peripheral lung lesion were measured by two radiologists; the presence of the bronchus sign was also recorded. Objective image noise was assessed using the standard deviation (SD) of the tracheal lumen attenuation by drawing a region of interest inside the trachea just proximal to the tracheal bifurcation area (Supplementary Figure S1) [12,13]. Final image noise was calculated by averaging the measurement values obtained by the two radiologists. Wall thickness and the wall-area ratio of the bronchus leading to the peripheral lung lesion were semi-automatically measured using 3D airway analysis software (Advantage Workstation 3.1; GE Healthcare). Peak wall thickness and the peak wall-area ratio were calculated from the reconstructed bronchi when the radiologist delineated a line along the bronchus leading to the peripheral lung lesion. The wall-area ratio was defined as the percentage of wall-area to total airway area at the reconstructed plane, orthogonal to the main axis of the bronchus. The bronchus sign was defined as the presence of a peripheral bronchus leading directly to peripheral lung lesions [14,15]. Two pulmonary physicians independently evaluated the bronchus sign on CT scans.

### 2.4. Statistical Analysis

All variables are presented as medians (interquartile range) and numbers (percentage) for continuous and categorical variables, respectively. Categorical variables were compared using Fisher’s exact test. The Kruskal–Wallis test or the Wilcoxon rank-sum test were used for continuous variables, as appropriate. Cohen’s kappa statistic was used for the measurement of inter-rater agreement for categorical variables. Differences with *p* < 0.05 were considered statistically significant. Statistical analyses were performed using SPSS for Windows, version 25.0 (IBM Corp., Armonk, NY, USA).

## 3. Results

**Figure 1:**
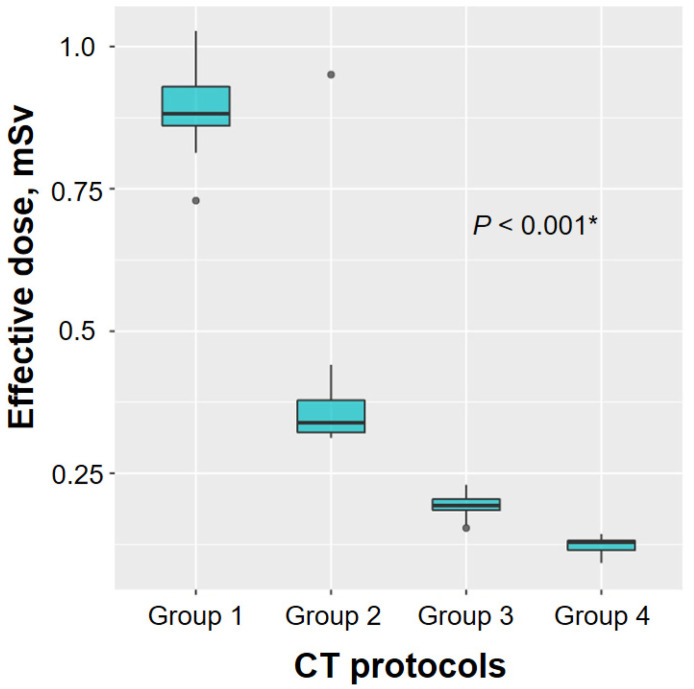
Comparison of the estimated effective ultra-low-dose computed tomography (CT) dose among the four study groups. Significant differences were observed in the median estimated effective dose of ultra-low-dose CT scans among the four groups (median estimated effective doses were 0.88, 0.34, 0.19, and 0.12 mSv for groups 1–4, respectively; *p* < 0.001). * Comparison for effective dose of ultra-low-dose CT among four groups.

In total, 91 consecutive patients with peripheral lung lesions were included in the study and assigned to the one of the four groups (25, 20, 24, and 22 patients in groups 1–4, respectively). The median patient age was 70 years (interquartile range, 61–75 years), and 55 patients (60%) were men (Supplementary Table S1). The median mean diameter of the lung lesions was 36 mm (interquartile range, 25–50 mm). The median estimated effective dose of the standard CT scan was 3.1 mSv (2.7–3.7), and the effective doses of the ultra-low-dose CT scans were significantly different among the four groups (median effective doses were 0.88, 0.34, 0.19, and 0.12 mSv for groups 1–4, respectively; *p* < 0.001) (Figure 1). Among 91 study patients, 72 received peripheral bronchoscopy and the diagnostic yield was 78% (Supplementary Table S2).

### 3.1. Objective Image Noise

Ultra-low-dose CT image noise was higher than image noise of standard-dose CT and increased significantly from group 1 to group 4 (*p* < 0.001 for both radiologists 1 and 2) (Table 1).

### 3.2. Bronchial Wall Thickness and Wall-Area Ratio

The difference in peripheral airway wall thickness was significantly higher in group 4 than in other groups (*p* = 0.011) in the analysis of radiologist 2 (Table 2). In the analysis of radiologist 1, the median difference in bronchial wall thickness in ultra-low-dose CT tended to be lower in groups 1–3 than in group 4; however, this difference was not statistically significant (0.5, 0.5, 0.5, and 0.9 mm for groups 1–4, respectively; *p* = 0.103). In the analysis of both radiologists 1 and 2, no significant differences in wall-area ratios were detected among the four ultra-low-dose CT protocols (*p* = 0.058 and 0.375 for radiologists 1 and 2, respectively) (Table 3).

### 3.3. Bronchus Sign

**Figure 2:**
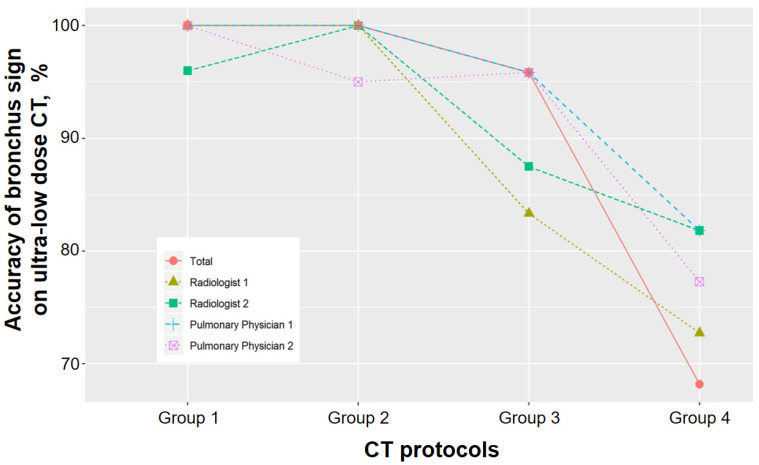
Comparison of the accuracy of bronchus sign on ultra-low-dose computed tomography (CT) among the four study groups.

**Figure 3:**
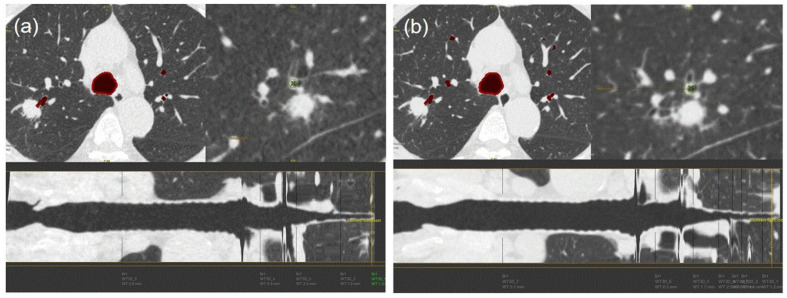
Airway analysis of a 63-year-old man with adenocarcinoma. The bronchus sign on an ultra-low-dose computed tomography (CT) scan (group 2, 100 kVp, 15 mAs) (**a**) completely corresponded with the bronchus sign of the standard CT scan (**b**). The differences in wall thickness and wall-area ratio between the standard and ultra-low-dose CT scans were 0.1 and 1.8, respectively.

In the analysis of radiologist 1, the bronchus sign of ultra-low-dose CT in groups 1 and 2 completely corresponded with that of standard CT; however, the accuracies of the bronchus sign of ultra-low-dose CT decreased to 83% and 73% in groups 3 and 4, respectively (*p* = 0.003) (Figure 2 and Figure 3). In the analysis of radiologist 2, the accuracies of the bronchus sign on ultra-low-dose CT in groups 3 and 4 tended to be lower than those of groups 1 and 2; however, these differences were not statistically significant (96%, 100%, 88%, and 82% for groups 1–4, respectively; *p* = 0.143) (Table 4). Agreement analysis between radiologists 1 and 2 showed a fair agreement for the identification of bronchus sign (agreement rate 89%, and Kappa coefficient 0.384).

The accuracies of the bronchus sign on ultra-low-dose CT in groups 1 and 2 were the same as those of standard-dose CT in the analysis of pulmonary physician 1, whereas accuracies decreased to 96% and 82% in groups 3 and 4, respectively (*p* = 0.017). In the analysis of pulmonary physician 2, a significant difference in the accuracy of bronchus sign was detected on ultra-low-dose CT among the four groups (*p* = 0.023). In particular, the bronchus sign of the ultra-low-dose CT was significantly lower in group 4 than in groups 1–3 (100%, 95%, 96%, and 77% for groups 1–4, respectively). Agreement analysis between pulmonary physicians 1 and 2 showed an excellent agreement for the identification of bronchus sign (agreement rate 98% and Kappa coefficient 0.822).

## 4. Discussion

Low-dose CT is generally used to screen for lung cancer in high-risk individuals, using minimal ionizing radiation, compared with a conventional chest CT scan [16,17]. Thus far, no study has been performed regarding the feasibility of low-dose CT for peripheral bronchoscopy. This is the first report in which the optimal effective dose CT scan has been evaluated for peripheral bronchoscopy without assistance of novel navigation modalities. In addition, this investigation used ultra-low-dose CT protocols with an estimated effective dose of <1 mSv.

In the present study, the ultra-low-dose CT protocols were designed to reduce the effective doses from group 1 to group 4. The results showed that the median estimated effective dose of ultra-low-dose CT gradually decreased from 0.88 mSv in group 1 to 0.12 mSv in group 4. Accordingly, the differences in image noise between standard-dose and ultra-low-dose CT, as measured by two radiologists, gradually increased from group 1 to group 4 (*p* < 0.001 for radiologists 1 and 2).

In general, reduction of the radiation dose inevitably increases image noise, which reduces image quality and spatial resolution. Therefore, bronchial walls are more likely to be found spread, and their margins are more likely to appear unclear when a lower effective dose is used. Our study showed that the visibility of the bronchial wall leading to the peripheral lung lesion decreased in group 4, compared with groups 1–3. Median differences in bronchial wall thickness between ultra-low-dose and standard-dose CT were 0.4–0.5 mm in groups 1–3. In addition, median differences in the wall-area ratio of the bronchus leading to the peripheral lung lesion were only 3–6% in groups 1–3.

The presence of a bronchus sign is reportedly a reliable predictor of a successful peripheral bronchoscopy procedure [7,18]. In the current study, the bronchus sign on ultra-low-dose CT in groups 1 and 2 was well correlated with the bronchus sign of a standard-dose CT scan. Our results suggested that both radiologists and pulmonary physicians could accurately identify the bronchus sign using reconstructed ultra-low-dose CT scans.

This study had several limitations. First, it was designed to focus solely on the interpretation of ultra-low-dose CT scans by radiologists and pulmonary physicians. Novel navigation modalities for bronchoscopy (e.g., virtual bronchoscopy navigation and electromagnetic navigation bronchoscopy) have been widely used to diagnose peripheral lung lesions under the guidance of artificial intelligence [19,20,21]. Therefore, bronchoscopy for peripheral lung lesions that depends solely on the interpretation of a CT scan by a doctor may be regarded as an outdated method. However, newer navigation modalities are not always 100% accurate [10,22]. In addition, navigation systems (e.g., electromagnetic navigation bronchoscopy) are quite expensive; thus, they are not available at all hospitals. Second, although all study patients were randomly assigned to one of the four groups and the database was updated prospectively, the current study was performed retrospectively with a relatively small study population at a single center. We acknowledge that potential selection bias might have influenced the results of our study. Third, 72 of 91 total study subjects received the bronchoscopy procedure for peripheral lung lesions and the diagnostic yield was 78%. However, the number of study subjects was too small to compare with the previous studies [23,24]. A randomized prospective study with a large number of patients is therefore needed to confirm our findings.

## 5. Conclusions

Our results indicate that ultra-low-dose CT with an effective dose of >0.34 mSv (effective dose of group 2) could be used for peripheral bronchoscopy instead of conventional CT scan.

## Figures and Tables

**Table 1:** Image noise of standard-dose computed tomography (CT) and ultra-low-dose CT.

	Group 1 (n = 25)	Group 2 (n = 20)	Group 3 (n = 24)	Group 4 (n = 22)	*p*-Value
Radiologist 1					
Image Noise on SCT	19 (17–20)	19 (18–22)	19 (17–21)	19 (18–22)	0.690
Image Noise on Ultra-LDCT	25 (24–27)	29 (28–32)	33 (32–34)	38 (36–46)	<0.001
Difference of Image Noise	6 (4–8)	11 (9–13)	14 (12–17)	18 (17–22)	<0.001
Radiologist 2					
Image Noise on SCT	20 (17–21)	20 (17–21)	19 (18–21)	18 (17–22)	0.990
Image Noise on Ultra-LDCT	27 (23–29)	31 (28–34)	34 (32–38)	37 (34–41)	<0.001
Difference of Image Noise	8 (5–10)	12 (10–14)	16 (13–19)	18 (14–21)	<0.001

Image noise was assessed using the standard deviation of tracheal lumen attenuation by drawing a region of interest inside the trachea just proximal to the tracheal bifurcation area. SCT = standard-dose CT, LDCT = low-dose CT. Image noise is presented as median (interquartile range).

**Table 2:** Wall thickness of the bronchus leading to the peripheral lung lesion.

	Group 1 (n = 25)	Group 2 (n = 20)	Group 3 (n = 24)	Group 4 (n = 22)	*p*-Value
Radiologist 1					
WT on SCT, mm	1.8 (1.3–2.6)	1.6 (1.3–2.0)	1.5 (1.2–2.2)	1.7 (1.3–2.2)	0.649
WT on ultra-LDCT, mm	1.7 (1.4–2.3)	1.7 (1.5–2.2)	2.0 (1.7–2.4)	2.5 (2.0–3.1)	0.018
Difference of WT	0.5 (0.3–0.9)	0.5 (0.3–0.7)	0.5 (0.3–0.6)	0.9 (0.5–1.4)	0.103
Radiologist 2					
WT on SCT, mm	1.9 (1.3–2.6)	1.7 (1.3–2.0)	1.5 (1.2–2.1)	1.6 (1.1–2.1)	0.322
WT on ultra-LDCT, mm	1.7 (1.4–2.1)	1.7 (1.4–1.9)	1.9 (1.7–2.4)	2.2 (1.7–3.1)	0.009
Difference of WT	0.5 (0.2–1.0)	0.5 (0.2–0.6)	0.4 (0.2–0.5)	0.8 (0.6–1.4)	0.011

WT = wall thickness, SCT = standard-dose CT, LDCT = low-dose CT. Wall thickness is presented as median (interquartile range).

**Table 3:** Wall-area ratio of the bronchus leading to the peripheral lung lesion.

	Group 1 (n = 25)	Group 2 (n = 20)	Group 3 (n = 24)	Group 4 (n = 22)	*p*-Value
Radiologist 1					
WAR on SCT, %	78 (73–84)	77 (76–81)	73 (71–79)	76 (73–80)	0.112
WAR on ultra-LDCT, %	79 (74–82)	75 (73–80)	77 (73–79)	78 (73–82)	0.505
Difference of WAR	5 (4–10)	3 (1–5)	4 (2–6)	7 (3–11)	0.058
Radiologist 2					
WAR on SCT, %	79 (75–84)	76 (75–81)	75 (71–78)	76 (73–81)	0.166
WAR on ultra-LDCT, %	79 (75–81)	77 (73–80)	77 (72–79)	78 (73–81)	0.553
Difference of WAR	5 (2–10)	4 (2–10)	6 (3–9)	10 (4–15)	0.375

WAR = wall-area ratio, SCT = standard-dose CT, LDCT = low-dose CT. Wall-area ratio is presented as median (interquartile range).

**Table 4:** Bronchus sign.

Accuracy of Bronchus Sign on Ultra-LDCT	Group 1 (n = 25)	Group 2 (n = 20)	Group 3 (n = 24)	Group 4 (n = 22)	*p*-Value
Radiologist 1	25/25 (100)	20/20 (100)	20/24 (83)	16/22 (73)	0.003
Radiologist 2	24/25 (96)	20/20 (100)	21/24 (88)	18/22 (82)	0.143
Pulmonary Physician 1	25/25 (100)	20/20 (100)	23/24 (96)	18/22 (82)	0.017
Pulmonary Physician 2	25/25 (100)	19/20 (95)	23/24 (96)	17/22 (77)	0.023

LDCT = low-dose computed tomography.

## Supplementary Materials

The following are available online at https://www.mdpi.com/1010-660X/56/9/479/s1, Figure S1: Measurement of objective image noise, Table S1: Patient demographics*, Table S2: Clinical diagnosis of 72 patients who received bronchoscopy.

## References

[1] Church T.R., Black W.C., Aberle D.R., Berg C.D., Clingan K.L., Duan F., Fagerstrom R.M., Gareen I.F., Gierada D.S., National Lung Screening Trial Research Team (2013). Results of initial low-dose computed tomographic screening for lung cancer. N. Engl. J. Med..

[2] Henschke C.I., Yankelevitz D.F., Mirtcheva R., McGuinness G., McCauley D., Miettinen O.S., ELCAP Group (2002). CT screening for lung cancer: Frequency and significance of part-solid and nonsolid nodules. AJR Am. J. Roentgenol..

[3] Detterbeck F.C., Lewis S.Z., Diekemper R., Addrizzo-Harris D., Alberts W.M. (2013). Executive Summary: Diagnosis and management of lung cancer, 3rd ed: American College of Chest Physicians evidence-based clinical practice guidelines. Chest.

[4] Lu C.H., Hsiao C.H., Chang Y.C., Lee J.M., Shih J.Y., Wu L.A., Yu C.J., Liu H.M., Shih T.T.F., Yang P.C. (2012). Percutaneous computed tomography-guided coaxial core biopsy for small pulmonary lesions with ground-glass attenuation. J. Thorac. Oncol..

[5] Gould M.K., Donington J., Lynch W.R., Mazzone P.J., Midthun D.E., Naidich D.P., Wiener R.S. (2013). Evaluation of individuals with pulmonary nodules: When is it lung cancer? Diagnosis and management of lung cancer, 3rd ed: American College of Chest Physicians evidence-based clinical practice guidelines. Chest.

[6] Kurimoto N., Miyazawa T., Okimasa S., Maeda A., Oiwa H., Miyazu Y., Murayama M. (2004). Endobronchial ultrasonography using a guide sheath increases the ability to diagnose peripheral pulmonary lesions endoscopically. Chest.

[7] Eom J.S., Mok J.H., Kim I., Lee M.K., Lee G., Park H., Lee J.W., Jeong Y.J., Kim W.Y., Jo E.J. (2018). Radial probe endobronchial ultrasound using a guide sheath for peripheral lung lesions in beginners. BMC Pulm. Med..

[8] Oki M., Saka H., Ando M., Asano F., Kurimoto N., Morita K., Kitagawa C., Kogure Y., Miyazawa T. (2015). Ultrathin bronchoscopy with multimodal devices for peripheral pulmonary lesions. A randomized trial. Am. J. Respir. Crit. Care Med..

[9] Ishida T., Asano F., Yamazaki K., Shinagawa N., Oizumi S., Moriya H., Munakata M., Nishimura M., Virtual Navigation in Japan Trial Group (2011). Virtual bronchoscopic navigation combined with endobronchial ultrasound to diagnose small peripheral pulmonary lesions: A randomised trial. Thorax.

[10] Eberhardt R., Anantham D., Ernst A., Feller-Kopman D., Herth F. (2007). Multimodality bronchoscopic diagnosis of peripheral lung lesions: A randomized controlled trial. Am. J. Respir. Crit. Care Med..

[11] McCollough C.H., Schueler B.A. (2000). Calculation of effective dose. Med. Phys..

[12] Boehm T., Willmann J.K., Hilfiker P.R., Weishaupt D., Seifert B., Crook D.W., Marincek B., Wildermuth S. (2003). Thin-section CT of the lung: Does electrocardiographic triggering influence diagnosis?. Radiology.

[13] Zhang L., Li Z., Meng J., Xie X., Zhang H. (2019). Airway quantification using adaptive statistical iterative reconstruction-V on wide-detector low-dose CT: A validation study on lung specimen. Jpn. J. Radiol..

[14] Gaeta M., Pandolfo I., Volta S., Russi E.G., Bartiromo G., Girone G., La Spada F., Barone M., Casablanca G., Minutoli A. (1991). Bronchus sign on CT in peripheral carcinoma of the lung: Value in predicting results of transbronchial biopsy. AJR Am. J. Roentgenol..

[15] Naidich D.P., Sussman R., Kutcher W.L., Aranda C.P., Garay S.M., Ettenger N.A. (1988). Solitary pulmonary nodules. CT-bronchoscopic correlation. Chest.

[16] Aberle D.R., Adams A.M., Berg C.D., Black W.C., Clapp J.D., Fagerstrom R.M., Gareen I.F., Gatsonis C., Marcus P.M., National Lung Screening Trial Research Team (2011). Reduced lung-cancer mortality with low-dose computed tomographic screening. N. Engl. J. Med..

[17] Pinsky P.F., Gierada D.S., Black W., Munden R., Nath H., Aberle D., Kazerooni E. (2015). Performance of Lung-RADS in the National Lung Screening Trial: A retrospective assessment. Ann. Intern. Med..

[18] Lee K.M., Lee G., Kim A., Mok J., Lee J.W., Jeong Y.J., Jo E.J., Kim M.H., Lee K., Kim K.U. (2019). Clinical outcomes of radial probe endobronchial ultrasound using a guide sheath for diagnosis of peripheral lung lesions in patients with pulmonary emphysema. Respir. Res..

[19] Mehta A.C., Hood K.L., Schwarz Y., Solomon S.B. (2018). The evolutional history of electromagnetic navigation bronchoscopy: State of the art. Chest.

[20] Chaddha U., Kovacs S.P., Manley C., Hogarth D.K., Cumbo-Nacheli G., Bhavani S.V., Kumar R., Shende M., Egan J.P., Murgu S. (2019). Robot-assisted bronchoscopy for pulmonary lesion diagnosis: Results from the initial multicenter experience. BMC Pulm. Med..

[21] Kemp S.V. (2020). Navigation Bronchoscopy. Respiration.

[22] Asano F., Matsuno Y., Shinagawa N., Yamazaki K., Suzuki T., Ishida T., Moriya H. (2006). A virtual bronchoscopic navigation system for pulmonary peripheral lesions. Chest.

[23] Steinfort D.P., Khor Y.H., Manser R.L., Irving L.B. (2011). Radial probe endobronchial ultrasound for the diagnosis of peripheral lung cancer: Systematic review and meta-analysis. Eur. Respir. J..

[24] Wang Memoli J.S., Nietert P.J., Silvestri G.A. (2012). Meta-analysis of guided bronchoscopy for the evaluation of the pulmonary nodule. Chest.

