# Supplementary material for: Feasibility of Ultra-Low-Dose CT for Bronchoscopy of Peripheral Lung Lesions
Source: Medicina (Kaunas). 2020 Sep 19;56(9):479. doi: 10.3390/medicina56090479 (PMC7559282; doi:10.3390/medicina56090479)
Supplement: Supplementary file 1 [file medicina-56-00479-s001.pdf]

Supplementary figure 1. Measurement of objective image noise.

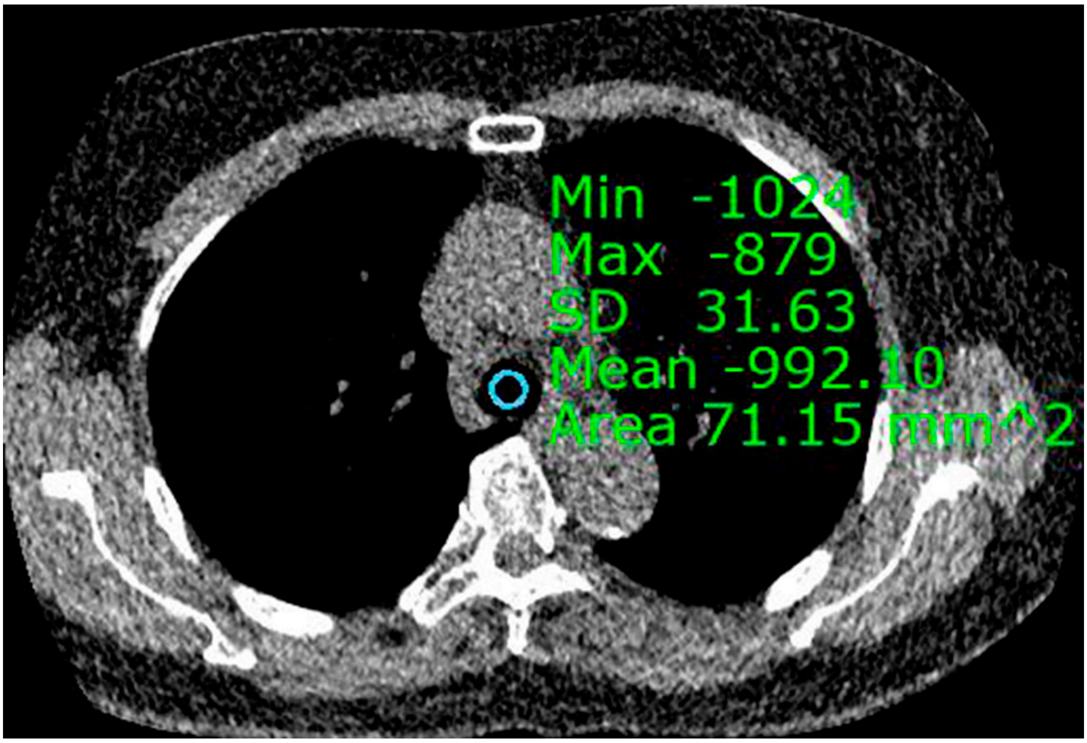

Supplementary table 1. Patient demographics*

	Group 1 (n = 24)	Group 2 (n = 20)	Group 3 (n = 24)	Group 4 (n = 23)	P-value
Age	64.5 (60.3-73.8)	69.0 (59.8-74.0)	71.5 (66.0-77.8)	68.0 (59.0-74.0)	0.159
Male gender	14 (58.3)	13 (65.0)	12 (50.0)	16 (69.6)	0.549
BMI	23.1 (20.8-24.7)	23.0 (20.8-25.0)	22.8 (20.8-23.9)	23.6 (21.5-25.8)	0.881

BMI = body mass index.

*Data was presented as numbers (%) or medians (interquartile range), as appropriate.

Supplementary table 2. Clinical diagnosis of 72 patients who received bronchoscopy.

Variables	No. (%)
Diagnosed with bronchoscopy	
Lung cancer	48 (67)
Lung metastasis from extrathoracic malignancy	4 (6)
Pulmonary tuberculosis	3 (4)
Organizing pneumonia	1 (1)
Undiagnosed with bronchoscopy	
Lung cancer	7 (10)
Lung metastasis from extrathoracic malignancy	1 (1)
Pulmonary tuberculosis	1 (1)
Non-tuberculous mycobacterial lung disease	1 (1)
Unknown	6 (8)
